# 液相色谱-串联质谱法测定泪液中维生素A和维生素E及其与干眼的关系分析

**DOI:** 10.3724/SP.J.1123.2025.06017

**Published:** 2026-03-08

**Authors:** Baobin LUO, Jingran JIAO, Jing BAI, Xiangyi LIU

**Affiliations:** 1.首都医科大学附属北京同仁医院，北京 100730; 1. Beijing Tongren Hospital，Capital Medical University，Beijing 100730，China; 2.首都医科大学附属北京佑安医院，北京 100069; 2. Beijing Youan Hospital，Capital Medical University，Beijing 100069，China

**Keywords:** 液相色谱-串联质谱, 泪液, 干眼, 维生素A, 维生素E, liquid chromatography-tandem mass spectrometry （LC-MS/MS）, tears, dry eye, vitamin A, vitamin E

## Abstract

泪液中维生素A（vitamin A， VA）和维生素E（vitamin E， VE）对于干眼的诊断和治疗具有潜在的临床预示价值，本研究建立了一种液相色谱-串联质谱法测定人泪液中VA和VE的分析方法。选用C18色谱柱（30 mm×2.1 mm， 2.6 μm），以0.1%甲酸水溶液-含2 mmol/L乙酸铵的0.1%甲酸甲醇溶液为流动相，采用大气压化学电离正离子模式，在0.4 mL/min的流速下进行梯度洗脱，4 min内可实现良好的色谱峰基线分离。经验证，该方法线性关系良好，VA的线性范围为5.00～300.00 ng/mL，检出限为2.00 ng/mL，加标回收率为98.3%～104.7%，日内精密度和日间精密度（以相对标准偏差（RSD）表示）为1.2%～7.0%；VE线性范围为25.00～1 000.00 ng/mL，检出限为6.00 ng/mL，加标回收率为97.9%～105.5%，日内精密度和日间精密度为3.0%～5.7%。使用本方法测定了5名干眼患者和9名健康志愿者的泪液标本，VA、VE质量浓度用中位数（四分位数间距）表示。干眼组泪液中VA含量为9.60（5.13～12.54） ng/mL，VE含量为42.00（31.75～128.00） ng/mL；健康组泪液中VA含量为18.10（12.46～21.69） ng/mL，VE含量为211.0（181.0～459.75） ng/mL。Wilcoxon Mann-Whitney两样本秩和检验干眼组和健康组泪液中VA、VE水平具有统计学差异，干眼组泪液中VA、VE浓度均低于健康组。本研究提供了一种简便可靠的泪液中VA、VE的液相色谱-串联质谱分析方法，探索了其与干眼的关系，为干眼相关因素的研究提供了新的可能的参考指标。

## Abstract (en)

Dry eye is characterized as a multifactorial disorder affecting the tear film and ocular surface， leading to symptoms of discomfort and visual disturbances， with the potential for ocular surface damage. It is among the most prevalent ocular surface disorders， with an estimated prevalence ranging from 5.0% to 50.0%， and an overall prevalence between 21.0% and 50.4% in China. Despite its high global prevalence， the fundamental mechanisms underlying the pathology of dry eye remain largely unexplored. Vitamins are crucial for maintaining ocular surface homeostasis， with vitamins A and E being physiologically present and essential for cell differentiation， development， and proper function. Deficiencies in these vitamins are frequently associated with dry eye， and systemic supplementation has been shown to benefit patients with low vitamin intake. Vitamins A and E may have potential clinical predictive value for the diagnosis and treatment of dry eye. In this study， a liquid chromatography-tandem mass spectrometry （LC-MS/MS） method was developed to simultaneously measure the concentrations of vitamins A and E in human tears. A Phenomenex Kinetex C18 column was employed for gradient elution at a flow rate of 0.4 mL/min. The mobile phase A consisted of a 0.1% formic acid aqueous solution， while mobile phase B comprised a 2 mmol/L ammonium acetate and 0.1% formic acid methanol solution. Positive atmospheric pressure chemical ionization in multiple reaction monitoring mode was utilized for MS/MS detection. The analytical method for vitamin A demonstrated a validated linear range of 5.00-300.00 ng/mL， with a limit of detection at 2.00 ng/mL. The spiked recoveries were between 98.3% and 104.7%， and intra- and inter-day precision ranged from 1.2% to 7.0%. Similarly， the method for vitamin E exhibited a validated linear range of 25.00-1 000.00 ng/mL， with a limit of detection at 6.00 ng/mL. The spiked recoveries ranged from 97.9% to 105.5%， and intra- and inter-day precision varied between 3.0% and 5.7%. Tear samples from five patients with dry eye and nine healthy volunteers were analyzed using this method. In the dry eye group， the average concentrations of vitamin A and vitamin E were 9.60 （5.13-12.54） ng/mL and 42.00 （31.75-128.00） ng/mL， respectively. In the healthy group， the average concentrations of vitamin A and vitamin E were 18.10 （12.46-21.69） ng/mL and 211.00 （181.00-459.75） ng/mL， respectively. Statistical differences in vitamin A and vitamin E levels between the dry eye group and the healthy group were identified using the Wilcoxon Mann-Whitney test （*P*<0.05）. This study introduces a straightforward and reliable LC-MS/MS method for the detection of tear VA and VE levels， while also investigating their association with dry eye. This offers a novel reference for understanding the potential risk factors associated with dry eye.

干眼（dry eye）为多因素引起的慢性眼表疾病，是由泪液的质、量及动力学异常导致的泪膜不稳定或眼表微环境失衡，可伴有眼表炎症反应、组织损伤及神经异常^［1］^。干眼常见的眼部症状包括眼干、眼红、异物感、畏光、分泌物增多、眼痒以及视疲劳，影响患者日常生活质量，严重者可导致角膜溃疡、穿孔，甚至失明^［2］^。干眼已成为眼科最常见的疾病之一，全球不同国家和地区干眼发病率为5%～50%，我国发病率为21%～52.4%^［3］^（数据来源于不同的流行病学调查）。干眼的诱发因素众多，发病机制复杂，其诊断方法很多，包括问卷量表、泪膜稳定性检测、泪液分泌量检测、眼表细胞染色以及眼影像学检查，但是多数方法均存在主观性强、缺乏客观量化指标的局限性^［4］^。

维生素在维持眼表微环境稳态中发挥重要作用。维生素A在角膜细胞的增殖、分化和生长过程中发挥重要的调节作用^［5］^，可以减轻眼表炎症，减少泪液蒸发，改善泪液质量^［6］^。维生素E为眼表提供抗氧化防御^［7］^。研究表明，维生素缺乏与干眼风险增加有关^［8］^，同时，补充维生素可以有效改善维生素缺乏患者干眼症状和体征^［9-11］^。

以往的有关维生素缺乏与干眼潜在关联的研究绝大多数是基于血液标本。泪液作为眼表微环境的重要组成部分，含有水、盐类、蛋白质、糖类、维生素等，作为一种无创性标本，有关泪液中维生素含量的研究较少，既往的为数不多的泪液维生素A和维生素E测定方法的研究采用的是高效液相色谱法（HPLC）^［12］^或液相色谱-串联质谱法（LC-MS/MS）^［13］^，相较于HPLC，LC-MS/MS具有更高的特异性和灵敏度，但之前LC-MS/MS的研究无法检测到泪液中维生素A，基于此，本研究拟建立一种泪液中维生素A和维生素E的LC-MS/MS检测方法，测定泪液中维生素A和维生素E的含量，并探讨其在干眼诊疗中应用的可能性。

## 1 实验部分

### 1.1 仪器、试剂与材料

#### 1.1.1 仪器与试剂

SCIEX Triple Quad^TM^ 4500MD液相色谱-串联质谱仪和Analyst 1.6.3数据处理软件（美国SCIEX公司）。色谱级甲醇、甲酸购自美国Fisher Scientific公司，维生素A、E标准品及其同位素内标维生素A-d4、维生素E-d6（纯度≥99.0%）购自美国Sigma公司，纯度≥99.0%。去离子水由PURELAB超纯水仪（英国ELGA公司）制备。

#### 1.1.2 泪液样本

征集5名首都医科大学附属北京同仁医院眼科确诊干眼志愿者。纳入标准：（1）年龄≥18岁。（2）干眼的诊断标准参考《中国干眼专家共识：检查和诊断（2020年）》^［4］^，①具有干眼相关症状，眼表疾病指数（ocular surface disease index， OSDI）≥5 s或非接触式泪膜破裂时间（non-invasive tear break-up time， NIBUT）<10 s或泪液分泌试验（Schirmer I试验）结果（无麻醉）≤5 mm/5 min；②具有干眼相关症状，OSDI≥13，荧光素染色泪膜破裂时间（fluorescein breakup time， FBUT）>5 s且≤10 s或NIBUT为10～12 s或Schirmer I试验结果（无麻醉）>5 mm/5 min且≤10 mm/5 min，荧光素染色法检查角膜和结膜呈阳性（染色点数≥5点）。排除标准：（1）合并神经性疼痛、过敏性结膜炎、干燥综合征、泪道梗阻疾病。（2）一个月内接受过干眼治疗或使用过隐形眼镜。（3）有眼部外伤史和手术史。另征集9名无干眼相关症状和历史的健康志愿者。采用毛细管法收集泪液标本，被采集者轻闭眼，头部稍后仰，将25 μL泪液采集毛细管一端以大约15°角轻触下睑缘中外1/3处的泪河下缘，利用毛细管的虹吸效应，泪液在表面张力作用下进入毛细管，采集至少12 μL泪液，置于1.5 mL EP管-80 ℃冰箱保存直至检测。本研究获得首都医科大学附属北京同仁医院医学伦理委员会的批准（TRECKY2021-090）。

### 1.2 实验方法

#### 1.2.1 标准溶液和内标溶液的配制

以甲醇为溶剂，配制质量浓度为5、10、20、40、100、300 ng/mL的维生素A和25、50、100、200、400、1 000 ng/mL的维生素E混合标准溶液。维生素A和维生素E内标用甲醇配制成终质量浓度为500 ng/mL的内标混合溶液。标准溶液和内标混合溶液分装于EP管中，-80 ℃保存。

#### 1.2.2 样本制备

将泪液标本、标准溶液和内标混合溶液取出解冻、混匀并恢复至室温。分别吸取10 μL标准溶液和泪液样品于1.5 mL EP管中，加入5 μL内标混合溶液混匀，涡旋振荡1 min，加入100 μL乙酸乙酯进行萃取，涡旋振荡10 min，4 800 r/min离心10 min。吸取80 μL上清液氮气吹干，加入20 μL乙腈进行复溶，涡旋振荡5 min，4 800 r/min离心5 min，取出全部上清液置于进样小瓶内插管中，上机进行检测。

#### 1.2.3 色谱条件

采用Phenomenex Kinetex C18色谱柱（30 mm×2.1 mm， 2.6 μm）。流动相A为0.1%甲酸水溶液，流动相B为含2 mmol/L乙酸铵的0.1%甲酸甲醇溶液。以0.4 mL/min的流速梯度洗脱，梯度如下：0～0.40 min，50%B；0.40～0.80 min，50%B～92%B；0.80～1.90 min，92%B～99%B；1.90～3.10 min，99%B；3.10～3.11 min，99%B～50%B；3.11～4.00 min，50%B。柱温40.0 ℃，进样量20 μL。

#### 1.2.4 质谱条件

大气压化学电离离子源，正离子模式（APCI^+^），离子源温度500 ℃，喷雾气379 kPa，气帘气138 kPa，碰撞气42 kPa，采用多反应检测扫描模式，各目标化合物和内标的质谱参数见表1。

**表1:** 目标化合物的质谱参数

Compound	Precursor ion （*m/z*）	Product ion （*m/z*）	DP/V	EP/V	CE/eV	CXP/V
Vitamin A （VA）	269.1	119.1^*^	50	10	45	6
		93.1	50	10	45	6
VA-d4	273.2	94.1	50	10	38	6
Vitamin E （VE）	431.2	165.2^*^	100	10	77	6
		137.0	100	10	77	6
VE-d6	437.1	171.4	100	10	27	6

* Quantitative ion. DP： declustering potential； EP： entrance potential； CE： collision energy； CXP： collision cell exit potential.

### 1.3 样本测定

用建立的方法测定5名干眼患者和9名健康志愿者泪液标本的VA和VE浓度，采用MedCalc20.0数据处理软件。Wilcoxon Mann-Whitney两样本秩和检验分析干眼组和健康组VA、VE水平是否有差异，定义*P<*0.05具有统计学差异**。**

## 2 结果与讨论

### 2.1 色谱条件的优化

流动相的选择直接影响目标化合物的色谱分离效果及质谱离子化效率，在流动相中加入适当的有机酸或者缓冲盐可以提高目标化合物的电离效率，提高分析方法的灵敏度。实验考察了水-甲醇、0.1%甲酸水-0.1%甲酸甲醇、0.1%甲酸水-含2 mmol/L乙酸铵的0.1%甲酸甲醇3种流动相对目标化合物离子化效率的影响，用0.1%甲酸水-含2 mmol/L乙酸铵的0.1%甲酸甲醇做流动相时，目标化合物的响应值均最高，且峰形尖锐，最终选择0.1%甲酸水-含2 mmol/L乙酸铵的0.1%甲酸甲醇做流动相。

### 2.2 质谱条件的优化

配制质量浓度为500 ng/mL的目标化合物混合标准溶液，对目标化合物及内标的质谱条件进行优化，包括母离子、子离子、去簇电压、碰撞能量等参数，全扫描模式下对化合物进行一级质谱分析，确定每种化合物的母离子，再通过对母离子进行二级质谱扫描优化碰撞能量，在优化后的碰撞能量下通过子离子扫描模式确定子离子。优化后的质谱条件见表1。

### 2.3 方法学验证

#### 2.3.1 总离子流图

由目标化合物的总离子流色谱图（图1）可见，在1.74 min和2.49 min处观察到两个明显的色谱峰，分别为VA和VE的色谱峰，目标峰形较为尖锐，附近未见到明显的影响积分的杂峰。

**图1:**
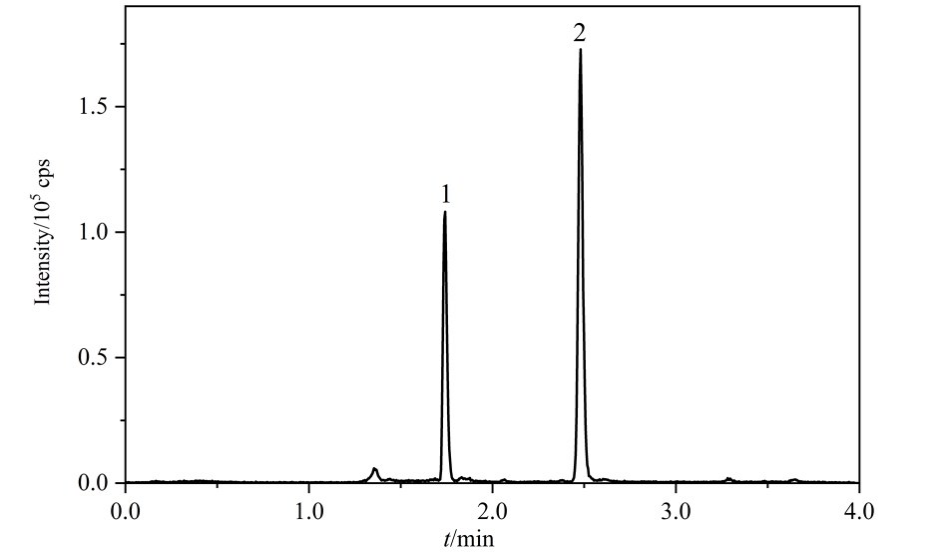
目标化合物的总离子流色谱图

#### 2.3.2 线性关系、检出限和定量限

参考刘朝阳等^［14］^的方法学验证方案，重复测定5次VA和VE系列混合标准溶液，以质量浓度为*X*，待分析物与内标峰面积的比值为*Y*，进行线性回归分析。稀释混合标准溶液，信噪比为3时的浓度定义为检出限（LOD）。稀释标准溶液，每个浓度重复测定20次，定量限（LOQ）应满足信噪比≥10，相对标准偏差（RSD）≤20%，测定均值与理论浓度偏差<15%。结果显示，5次实验的线性相关系数（*R* ^2^）大于0.999，表明线性关系良好，VA线性范围为5.00～300.00 ng/mL，VA检出限为2.00 ng/mL。VE线性范围为25.00～1 000.00 ng/mL，检出限为6.00 ng/mL，结果见表2。

**表2:** 目标化合物的线性方程、相关系数、线性范围、检出限和定量限

Compound	Linear equation	*R* ^2^	Linear range/（ng/mL）	LOD/（ng/mL）	LOQ/（ng/mL）
VA	*Y*=0.01437*X*+0.16268	0.9997	5.00-300.00	2.00	5.00
VE	*Y*=0.00381*X-*0.00306	0.9990	25.00-1000.00	6.00	25.00

*Y：* peak area ratio of analyte to internal standard； *X*： mass concentration of VA or VE， ng/mL.

#### 2.3.3 回收率和精密度

分别向两种泪液标本中加入30.00 ng/mL、100.00 ng/mL的VA标准溶液和100.00 ng/mL、300.00 ng/mL的VE标准溶液，每种加标样本平行制备5份，重复测定5个批次。测定加标前后的样本中VA、VE的含量，计算目标物的回收率及相对标准偏差，结果表明VA加标回收率为98.3%～104.7%，VE加标回收率为97.9%～105.5%。

连续测定5天，每天一个分析批，每批两个浓度水平，每一个浓度水平同一样本重复测定5次。VA日内精密度和日间精密度RSD为1.2%～7.0%，VE日内精密度和日间精密度RSD为3.0%～5.7%，结果见表3。

**表3:** 目标化合物在2个水平下的加标回收率和精密度（*n*=5）

Analyte	Background/ （ng/mL）	Added/ （ng/mL）	Detected/ （ng/mL）	Found/ （ng/mL）	Recovery/%	Intra-day RSD/%	Inter-day RSD/%
VA	14.48	30.00	45.13	30.65	102.2	1.9	7.0
		100.00	112.82	98.34	98.3	1.3	3.4
	15.80	30.00	47.20	31.40	104.7	2.1	6.9
		100.00	115.10	99.30	99.3	1.2	3.5
VE	260.00	100.00	365.50	105.50	105.5	3.0	5.5
		300.00	558.30	298.30	99.4	2.9	4.5
	290.00	100.00	392.00	102.00	102.0	3.2	5.7
		300.00	583.80	293.80	97.9	3.1	4.5

### 2.4 方法的人群应用

采集5例临床诊断为干眼患者的泪液标本，同时采集9例性别、年龄相匹配的健康志愿者泪液标本，干眼组（dry eye group， DG）和健康组（healthy group， HG）的基本情况（性别、年龄、泪液中VA和VE浓度）信息见表4，HG组9名志愿者泪液VA、VE浓度均在所建方法线性范围内，DG组5名干眼患者中，4名泪液VA、VE含量在线性范围内，1名干眼患者VA、VE均小于定量限，在进行中位数（四分位数间距）描述和Wilcoxon Mann-Whitney秩和检验时，将该名干眼患者的VA、VE含量按照定量限处含量处理。将泪液VA、VE含量用中位数（四分位数间距）［Median（P_25_~P_75_）］表示，干眼组的VA含量为9.60（5.80～12.54）ng/mL，健康组VA含量为18.10（12.46～21.69）ng/mL，干眼组的VE含量为42.00（31.75～128.00）ng/mL，健康组VE含量为211.00（181.00～459.75）ng/mL。Ubels等^［12］^应用液相色谱法检测到正常成人泪液中VA的含量约为16 ng/mL，与本研究中健康组VA含量水平较为接近。Khaksari等^［13］^应用LC-MS/MS检测到成人泪液中VE的质量含量为14.2～408.5 ng/mL，但并未对研究人群健康状态进行描述。

**表4:** 受试者基本情况

Group	Age/year	Sex	VA/（ng/mL）	VE/（ng/mL）
HG-1	32	female	18.06	448.00
HG-2	31	male	20.39	211.00
HG-3	39	female	12.00	182.00
HG-4	34	male	14.18	178.00
HG-5	37	male	24.07	495.00
HG-6	41	female	22.44	521.00
HG-7	42	male	12.34	193.00
HG-8	39	male	21.45	420.00
HG-9	43	male	12.50	176.00
DG-1	40	male	10.77	95.00
DG-2	36	male	6.07	34.00
DG-3	30	female	<5.00	<25.00
DG-4	43	male	17.84	42.00
DG-5	31	female	9.60	227.00

HG： healthy group； DG： dry eye group.

经Wilcoxon Mann-Whitney两样本秩和检验，干眼组和健康组VA含量差值中位数（median difference）为7.34 ng/mL，95%置信区间（confidence interval， CI）为（1.73～14.48） ng/mL，干眼组和健康组VE含量差值中位数为169.00 ng/mL，95%CI为（87.00～423.00） ng/mL。干眼组泪液VA和VE含量均低于健康组，差异有统计学意义（*P*<0.05），结果见表5。

**表5:** 干眼组和健康组泪液中VA、VE含量比较

Analyte	DG^1）^/（ng/mL）	HG^1）^/（ng/mL）	Median difference ^1）2）^（95%CI）/（ng/mL）	*Z*	*P*
VA	9.60 （5.13， 12.54）	18.10 （12.46， 21.69）	7.34 （1.73， 14.48）	2.46	0.014
VE	42.00 （31.75， 128.00）	211.00 （181.00， 459.75）	169.00 （87.00， 423.00）	2.33	0.019

1） Median （P_25_， P_75_）； 2） Median difference=HG-DG.

既往的干眼泪液生物标志物的研究显示，泪液中潜在的干眼相关标志物包括细胞因子^［15］^、生长因子^［16］^、细胞黏附分子及可溶性受体^［17］^等。本研究基于横断面研究发现干眼组和健康组泪液中VA、VE含量存在统计学差异，但因样本量较小，后续尚需进一步扩大样本量，优化研究设计，进一步证实泪液中VA、VE和干眼的相关性。

## 3 结论

本研究建立了一种基于液相色谱-串联质谱法的泪液VA、VE检测方法，该方法快速、简便、精密，能有效分离和测定泪液中VA、VE，为临床监测人群泪液VA、VE水平提供了一个新方法，具有潜在的应用价值，为泪液VA、VE水平与干眼之间的相关性研究提供了新的参考。
